#  “SILVAMP TB LAM” Rapid Urine Tuberculosis Test Predicts Mortality in Patients Hospitalized With Human Immunodeficiency Virus in South Africa

**DOI:** 10.1093/cid/ciaa024

**Published:** 2020-01-09

**Authors:** Bianca Sossen, Tobias Broger, Andrew D Kerkhoff, Charlotte Schutz, Andre Trollip, Emmanuel Moreau, Samuel G Schumacher, Rosie Burton, Amy Ward, Robert J Wilkinson, David A Barr, Mark P Nicol, Claudia M Denkinger, Graeme Meintjes

**Affiliations:** 1 Wellcome Centre for Infectious Diseases Research in Africa, Institute of Infectious Disease and Molecular Medicine, University of Cape Town, Republic of South Africa; 2 Department of Medicine, Faculty of Health Sciences, University of Cape Town, Republic of South Africa; 3 Foundational for Innovative Diagnostics, Geneva, Switzerland; 4 Division of HIV, Infectious Diseases and Global Medicine at Zuckerberg San Francisco General Hospital and Trauma Center, Department of Medicine, University of California, San Francisco, California, USA; 5 Southern African Medical Unit, Médecins sans Frontières, Cape Town, South Africa; 6 The Francis Crick Institute, London, United Kingdom; 7 Department of Medicine, Imperial College, London, United Kingdom; 8 Wellcome Trust Liverpool Glasgow Centre for Global Health Research, University of Liverpool, United Kingdom; 9 Division of Infection and Immunity, School of Biomedical Sciences, University of Western Australia, Perth, Australia; 10 Division of Medical Microbiology, University of Cape Town, Republic of South Africa; 11 Division of Tropical Medicine, Center of Infectious Diseases, University of Heidelberg, Heidelberg, Germany

**Keywords:** Tuberculosis, HIV, lipoarabinomannan, point-of-care, mortality

## Abstract

Reducing diagnostic delay is key toward decreasing tuberculosis-associated deaths in people living with human immunodeficiency virus. In tuberculosis patients with retrospective urine testing, the point-of-care Fujifilm SILVAMP TB LAM (FujiLAM) could have rapidly diagnosed tuberculosis in up to 89% who died. In FujiLAM negative patients, the probability of 12-week survival was 86–97%.

Tuberculosis (TB) is the leading infectious cause of death worldwide and contributed to approximately 300 000 deaths related to human immunodeficiency virus (HIV) in 2017. Diagnostic delay remains a key factor contributing to mortality in HIV-associated TB[1]. Xpert MTB/RIF (Xpert, Cepheid) and Xpert MTB/RIF Ultra are the first-line tests recommended by the World Health Organization (WHO) to diagnose HIV-associated TB. Although they provide a sensitive diagnosis, they are often unavailable at point-of-care (POC), and patients are not always able to provide sputum[2, 3]. The urine-based Alere Determine-TB lipoarabinomannan (LAM) assay (AlereLAM, Abbott) was the first POC assay for TB, and its use has been shown to reduce all-cause mortality in high-risk groups of people living with HIV (PLHIV), in 2 randomized trials[4, 5]. Although AlereLAM has been recommended by the WHO in high-risk groups, its uptake has been constrained—partially due to limited diagnostic sensitivity.

We recently published findings on a next-generation urine LAM assay, Fujifilm SILVAMP TB LAM (FujiLAM), demonstrating its superior sensitivity and similar specificity to AlereLAM in hospitalized PLHIV [6]. FujiLAM’s high sensitivity is achieved through a pair of high-affinity monoclonal antibodies [7] and a silver amplification step. In the identical cohorts presented here, we have also demonstrated that FujiLAM achieves good sensitivity for detecting both pulmonary and extra-pulmonary TB [8]. Here we assessed FujiLAM’s ability to predict mortality in these 2 well-characterized cohorts of PLHIV being evaluated for TB and requiring hospitalization.

## METHODS

For this *post hoc* mortality analysis, we utilized outcome data from 2 prospective cohorts of the previously published diagnostic accuracy study [6]. Both studies enrolled adult (⩾18years) PLHIV requiring hospitalization at South African district hospitals, serving communities with a high burden of HIV-associated TB. “Cohort A” consecutively enrolled all consenting PLHIV admitted to the adult medical wards at GF Jooste Hospital, irrespective of CD4 count or clinical suspicion of TB, between June 2012 and October 2013[9]. “Cohort B” enrolled hospitalized adult PLHIV at Khayelitsha Hospital with CD4 cell counts ≤ 350 cells/µL in whom TB was considered to be the most likely diagnosis, from January 2014 to October 2016 [10]. Both cohorts excluded those already on TB treatment at the time of admission. The study was approved by the Human Research Ethics Committee of the University of Cape Town (UCT). Written informed consent was obtained from patients, as per study protocols.

The demographic and clinical details of patients were recorded at study entry. TB or alternative diagnoses were based on a comprehensive laboratory work-up including Xpert and/or mycobacterial culture in sputum, blood, urine, and other clinically indicated samples, along with clinical examination and follow-up, as previously described [6, 9, 10] (Supplementary Table 1). FujiLAM and AlereLAM testing was performed on thawed, biobanked urine samples at UCT in April 2018[6], according to manufacturer’s instructions. “Microbiologically confirmed TB” was defined by the detection of *Mycobacterium tuberculosis* in any clinical specimen using culture or Xpert. In Cohort A, “clinically confirmed TB” was defined as those without a microbiological diagnosis of TB but where the nonstudy clinical team had started TB treatment based on clinical and/or radiological features of TB, as per the primary diagnostic accuracy study [6]. In Cohort B, “clinically confirmed TB” could not be defined as per the primary diagnostic study [6], as this reference standard incorporated survival criteria. Therefore, a composite clinical reference standard was used for “clinically confirmed TB” in Cohort B that used clinical, radiological, laboratory, and treatment data to determine clinical TB diagnoses [10]. AlereLAM and FujiLAM results were not included within the reference standards. “TB patients” refers to those with either “microbiologically confirmed TB” or “clinically confirmed TB.” Analyses that were limited to microbiologically confirmed TB are specified as such. Twelve-week (84-day) outcomes for all patients (alive, dead, or lost to follow-up) were determined from case notes, health record databases, telephone calls, or personal visits [9, 10].

The proportion of TB patients who died who could have been rapidly diagnosed by each LAM assay with or without the addition of sputum Xpert was calculated. Kaplan-Meier plots were generated to investigate the cumulative mortality risk by FujILAM and AlereLAM test result. Negative predictive values (NPV) for death with Wilson 95% confidence intervals (CIs) were calculated for each LAM assay, by dividing the total number of LAM-negative survivors at 12 weeks by the total number of LAM-negative patients. The NPV represents the probability that those with a negative LAM result survive 12 weeks. Whether patients were treated for TB or any delays in treatment initiation were not factored into outcome analyses. Analysis was done using R (version 3.6.0).

## RESULTS

Overall, 983 patients were included in this analysis: 410 from Cohort A and 573 from Cohort B (Supplementary Figure 1). The median age of patients in both cohorts was 36 years, the majority were female, and nearly half had a prior history of TB (Supplementary Table 2). There were notable differences between Cohorts A and B due to the study eligibility criteria: Cohort B had lower median CD4 counts, a higher proportion of microbiologically confirmed TB, and features of more advanced illness.

Among TB patients, 18/175 (10.3%) in Cohort A and 101/496 (20.4%) in Cohort B died within 12 weeks. In TB patients who died in Cohort A, FujiLAM could have diagnosed TB in 88.9% (n = 16/18; 95% CI: 67.2–96.9) versus 66.7% (n = 12/18; 95% CI: 43.7–83.7) by AlereLAM (Supplementary Figure 2A). All TB patients who died in Cohort A were also microbiologically confirmed. Sputum Xpert testing would not have increased the diagnostic yield in this group.

In TB patients who died in Cohort B, FujiLAM could have rapidly diagnosed TB in 80.2% (n = 81/101; 95% CI: 71.4–86.8) versus 43.6% (n = 44/101; 95% CI: 34.3–53.3) by AlereLAM (Supplementary Figure 2B). The combination of FujiLAM and sputum Xpert could have diagnosed 83.2% (n = 84/101) of TB patients who died and 96.5% (n = 82/85) of microbiologically confirmed TB patients who died in Cohort B. The combination of AlereLAM and sputum Xpert could have diagnosed 69.3% (n = 70/101) of TB patients who died and 80.0% (n = 68/85) of microbiologically confirmed TB patients who died in Cohort B (Supplementary Figure 2B).

**Figure 1.**
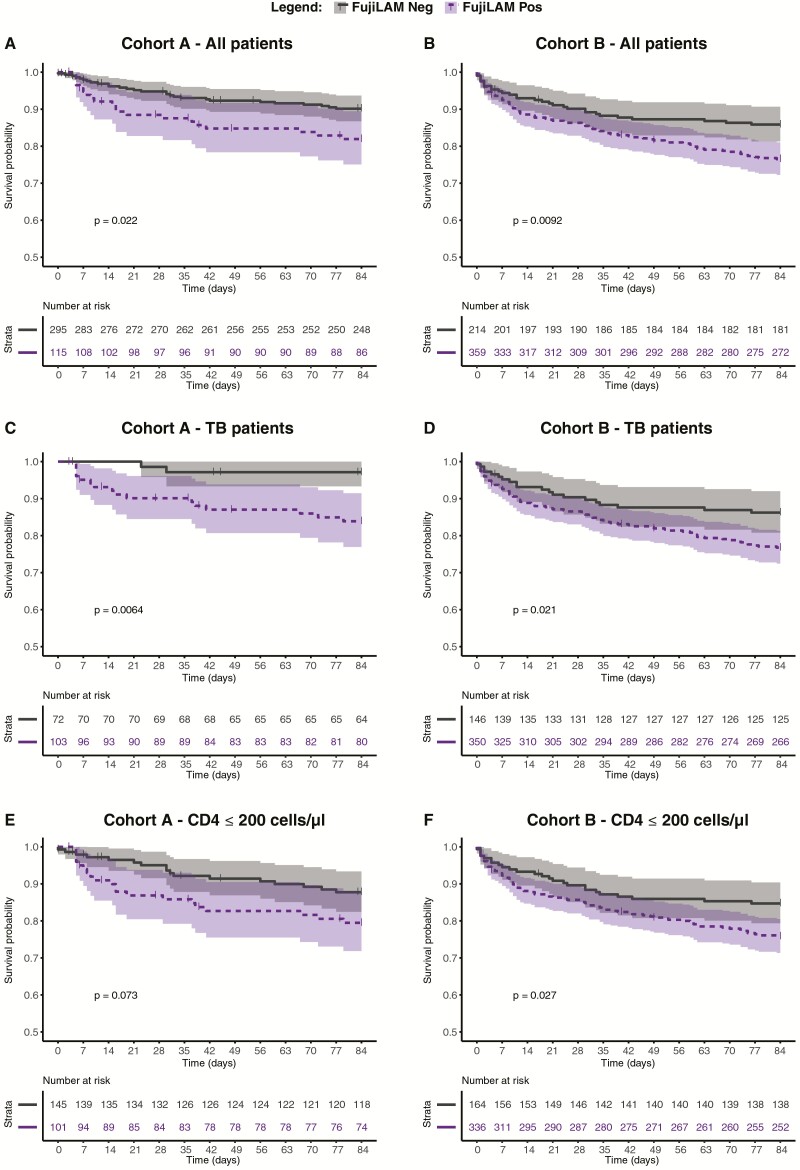
Kaplan Meier survival curves by FujiLAM status, up to 12 weeks of follow-up. All patients of Cohort A (n = 410) (***A***) and Cohort B (n = 573) (***B***). TB patients from Cohort A (n = 175) (***C***), and Cohort B (n = 496) (***D***). Patients with CD4 ≤ 200 cells/µL, irrespective of TB diagnosis from Cohort A (n = 246) (***E***) and Cohort B (n = 500) (***F***). *P* values are based on log-rank test and bands represent 95% confidence intervals. Abbreviation: TB, tuberculosis.

Kaplan-Meier mortality curves stratified by FujiLAM-status for all patients in Cohorts A and B, and by subgroups, are shown in Figure 1. In both cohorts, a positive FujiLAM was associated with all-cause mortality in the entire cohort up to 12 weeks (Figure 1A and 1B), when limiting to TB patients (Figure 1C and 1D) and when limiting to those with CD4 counts ≤ 200cells/µL (independent of TB diagnosis) (Figure 1E and 1F). In contrast, an association between AlereLAM result and mortality was only seen in Cohort A, among all patients (Supplementary Figure 3A) and its TB patients (Supplementary Figure 3C).

When assessing the NPV of FujiLAM for 12-week mortality, the probability of survival to 12 weeks was 89.9% (95% CI: 85.7–92.9) in unselected FujiLAM-negative patients from Cohort A and 97.0% (95% CI: 89.6–99.2) in the TB patients of Cohort A (Supplementary Figure 4). The probability of survival to 12 weeks with a negative FujiLAM was 85.8% (95% CI: 80.4–89.9) in all patients from Cohort B and was 86.2% (95% CI: 79.7–90.9) in the TB patients of Cohort B. Results for AlereLAM and for both LAM assays in microbiologically confirmed TB are presented in Supplementary Figure 4.

## Discussion

In this *post hoc* analysis of 2 South African cohorts of hospitalized PLHIV, we found that FujiLAM would have rapidly detected TB in up to 89% of patients who died within 3 months. Furthermore, in TB patients, a negative FujiLAM result was associated with between 86 and 97% probability of survival to 3 months.

AlereLAM-based diagnosis in combination with rapid TB treatment initiation has already been shown to reduce mortality in high-risk PLHIV and the association between detection of urinary LAM and mortality is not novel [4, 5]. However, our findings suggest that the improved sensitivity of FujiLAM to diagnose TB, including in less immunocompromised individuals[6], may further increase positive impact on survival by enabling rapid, POC diagnosis of TB in a larger proportion of patients. Compared to AlereLAM, FujiLAM detected TB in 22–37% more PLHIV who died. FujiLAM shows great promise as an initial TB test allowing for rapid treatment initiation in more patients, WHILE drug susceptibility is confirmed. Those testing FujiLAM-negative have a reassuring low risk of short-term mortality, with high NPV for death, suggesting less need for immediate empiric TB treatment in this group—they should undergo further rapid tests, such as the Xpert, to establish an expedited diagnosis in the majority.

Strengths of this study include analysis of 2 well-characterized large cohorts, with low rate of loss-to-follow-up. With retrospective urine testing, we cannot assess the real-world feasibility of FujiLAM, or whether it could have changed TB treatment practices or patient survival. Although the results suggest that there is room for benefit due to improved sensitivity, we also recognize the slightly lower specificity noted in our previous study with the FujiLAM assay, even though this difference was not statistically significant and might be a result of the imperfect reference standard [6]. The risk of treatment based on potentially false-positive results should be weighed against the risk of not treating hospitalized PLHIV at high risk of mortality due to TB.

Our results suggest that in addition to the superior sensitivity of FujiLAM in comparison to AlereLAM, FujiLAM also has greater prognostic value. The clinical impact, including the potential survival benefit of FujiLAM, should be prospectively assessed in different real-world settings and more diverse populations—including outpatients, children, and HIV-uninfected patients.

## Supplementary Data

Supplementary materials are available at *Clinical Infectious Diseases* online. Consisting of data provided by the authors to benefit the reader, the posted materials are not copyedited and are the sole responsibility of the authors, so questions or comments should be addressed to the corresponding author.

ciaa024_suppl_Supplementary_Material

## Notes

***Authors Contributions*.** T. B., B. S., A. D. K., C. S., M. P. N., G. M., and C. M. D. designed the study, and T. B., B. S., A. T., M. P. N., G. M., and C. M. D. oversaw the study. B. S., A. D. K., C. S., A. W., D. A. B., R. B., M. P. N., R. J. W., and G. M. coordinated the individual study sites. B. S., T. B., and A. D. K. did the statistical analysis and wrote the first manuscript draft. All authors contributed to interpretation of data and editing of the article and approved the final version of the manuscript.

***Acknowledgments.*** The authors acknowledge the late Stephen D Lawn who designed and led the Cohort-A study. The authors thank the clinical and laboratory teams at the partner sites for their efforts in the implementation, conduct, and timely completion of the study. In particular, we thank Judi van Heerden, Nchimunya Hapeela, Elloise du Toit, Amanda Jackson, Celeste Worship, Meagan McMaster, Rita Szekely, Michelle Bulterys, and Aurélien Mace.

***Disclaimer*.** The funders had no role in the study design, data collection, data analysis, data interpretation, or writing of this report. The opinions, findings, and conclusions expressed in this manuscript reflect those of the authors alone.

***Financial support*.** This work was supported by the Global Health Innovative Technology Fund [grant numbers G2015-201, G2017-207]; the UK Department for International Development [300341-102]; the Dutch Ministry of Foreign Affairs [PDP15CH14]; the Bill and Melinda Gates Foundation [OPP1105925]; the Australian Department of Foreign Affairs and Trade [70957]; the German Federal Ministry of Education and Research through KfW; Wellcome [088590, 085251, 098316, 203135/Z/16/Z, 088316], the Francis Crick Institute which receives funding from Wellcome [FC0010218 to RJW]; the South African Research Chairs Initiative of the Department of Science and Technology and National Research Foundation of South Africa [64787 to G. M.]; the South African Medical Research Council with funds received from the National Department of Health [RFA#SAMRC-RFA-CC:TB/HIV/AIDS-01-2014 to G. M.]; Cancer Research UK [FC0010218 to R. J. W.]; UK Research and Innovation [FC0010218 to R. J. W.]; the National Institutes of Health [U01AI 115940 to R. J. W., T32 AI060530 to A. D. K.]; and the National Health Scholarship Programme of the South African Medical Research Council [to C. S.].

***Potential conflicts of interest.*** T. B. and C. D. were previously employed by Foundation for Innovative Diagnostics (FIND), and A. T., E. M., and S. S. are currently employed by FIND. FIND is a not-for-profit foundation that supports the evaluation of publicly prioritized tuberculosis assays and the implementation of WHO-approved (guidance and prequalification) assays using donor grants. FIND has product evaluation agreements with several private sector companies that design diagnostics for tuberculosis and other diseases. These agreements strictly define FIND’s independence and neutrality vis-à-vis the companies whose products get evaluated and describe roles and responsibilities. M. N. reports grants from FIND during the conduct of the study. R. J. W. reports grants from European and Developing Countries Clinical Trials Partnership, during the conduct of the study. T. B. reports a patent in the field of lipoarabinomannan detection. All other authors report no potential conflicts. All authors have submitted the ICMJE Form for Disclosure of Potential Conflicts of Interest. Conflicts that the editors consider relevant to the content of the manuscript have been disclosed.
