# Supplementary material for: “SILVAMP TB LAM” Rapid Urine Tuberculosis Test Predicts Mortality in Patients Hospitalized With Human Immunodeficiency Virus in South Africa
Source: Clin Infect Dis. 2020 Jan 9;71(8):1973–6. doi: 10.1093/cid/ciaa024 (PMC8240995; doi:10.1093/cid/ciaa024)
Supplement: ciaa024_suppl_Supplementary_Material [file ciaa024_suppl_supplementary_material.docx]

**Supplementary material: ‘SILVAMP TB LAM’ rapid urine tuberculosis test predicts mortality in hospitalized HIV patients in South Africa**

**Supplementary Table 1:** Definitions of microbiologically-confirmed and clinically-confirmed TB patients, per cohort.

| **Cohort** | **Microbiologically-confirmed TB** | **Clinically-confirmed TB** |
| --- | --- | --- |
| Cohort-A | Any culture or any Xpert positive for *M.tuberculosis* from any available anatomic sample | Any patient not meeting ‘microbiologically-confirmed’ TB definition who has either:   1. clinical/radiographic features suggestive of TB   OR   1. any patient started on TB treatment |
| Cohort-B | Any culture or any Xpert positive for *M.tuberculosis* from any available anatomic sample | Any patient not meeting ‘microbiologically-confirmed’ TB definition who has any of the following:   1. Pleural effusion which was treated for tuberculosis or exudative pleural effusion with adenosine deaminase >30g/dl and no alternative diagnosis made 2. Pericardial effusion treated for tuberculosis and no alternative diagnosis made 3. Miliary tuberculosis on chest X-ray, treated for tuberculosis and no alternative diagnosis made 4. Features of tuberculosis on abdominal ultrasound (multiregion nodes ≥ 1cm diameter or splenic microabscesses) treated for tuberculosis and no alternative diagnosis made 5. Cerebrospinal fluid (CSF) picture compatible with probable tuberculous meningitis (TBM) with CSF score ≥2 out of 4^[1]^, treated for TBM and no alternative diagnosis made 6. Computed tomography (CT) scan features of central nervous system tuberculosis, treated for tuberculosis with no alternative diagnosis made 7. Chest X-ray compatible with tuberculosis, treated for tuberculosis and remained on treatment |

**[1]** Marais S, Thwaites G, Schoeman JF, et al. Tuberculous meningitis: a uniform case definition for use in clinical research*. Lancet Infect Dis* 2010; 10(11): 803-12.

**Supplementary Table 2:** Descriptive characteristics of participants, per cohort, at baseline

|  | Cohort-A  (n=410) | Cohort-B  (n=573) |
| --- | --- | --- |
| **Age**, median (IQR), years | 36 (29 – 42) | 36 (31 – 44) |
| **Females**, n (%) | 248 (60.5) | 293 (51.1) |
| **Known TB history**, n (%) | 185 (45.1) | 262 (45.7) |
| **ART status** |  |  |
| - Naive | 174 (42.4) | 220 (38.8) |
| - ART interrupted | 64 (15.6) | 138 (24.3) |
| - Currently on ART | 172 (42.0) | 209 (36.9) |
| **CD4 count**, cells/ul |  |  |
| - median (IQR) | 150 (56 – 311) ^a^ | 63 (24 – 131) |
| - ≤ 200, n (%) | 246 (60.3) ^a^ | 500 (87.3) |
| - > 200, n (%) | 162 (39.7) ^a^ | 73 (12.7) |
| **Haemoglobin**, g/dl |  |  |
| - median (IQR) | 9.7 (7.7 – 11.6) ^b^ | 8.9 (7.3 – 10.6) |
| - severe anaemia (Hb<8g/dl), n (%) | 120 (29.7) ^b^ | 197 (34.4) |
| **CRP**, median (IQR) | 73.1 (17.2 – 158.8) ^c^ | 104 (81 – 142) ^d^ |
| **TB reference category** |  |  |
| - Microbiologically-confirmed TB, n (%) | 138 (33.7) | 415 (72.4) |
| - Clinically-confirmed TB, n (%) | 37 (9.0) | 81 (14.1) |
| - Not TB, n (%) | 189 (46.1) | 42 (7.3) |
| - Unclassifiable, n (%) | 46 (11.2) | 35 (6.1) |
| **Site of Microbiologically-confirmed TB ^e,f^** |  |  |
| - Confirmed on sputum Xpert or Culture, n (%) | 74 (53.6) | 323 (77.8) |
| - Confirmed on blood Cultre, n (%) | 40 (30.0) | 180 (43.4) |
| - Confirmed on urine Xpert or Culture, n (%) | 91 (65.9) | 222 (53.5) |
| - Confirmed on cerebrospinal fluid Xpert or Culture, n (%) | 7 (5.1) | 7 (1.7) |
| - Confirmed on pleural fluid Xpert or Culture, n (%) | 13 (9.4) | 46 (11.1) |
| - Confirmed in other extrapulmonary site Xpert or Culture, n (%) | 12 (8.7) | 19 (4.6) |
| **TB treatment** |  |  |
| - Initiated TB treatment | 133 (32.4) | 510 (89.0) |
| - No TB treatment initiated or unknown | 277 (67.6) | 63 (11.0) |
| **Outcome at 12 weeks** |  |  |
| - Died | 48 (11.7) | 114 (20.0) |
| - Survived | 334 (81.5) | 453 (79.1) |
| - Lost to follow-up | 28 (6.8) | 6 (1.1) |
| ^a^ 2 missing data points; ^b^ 6 missing data points; ^c^ 19 missing data points; ^d^ 3 missing data points; ^e^ Groups of the sites of microbiologically-confirmed TB are not mutually exclusive i.e. patients can contribute to multiple groups; ^f^ patients were not systematically sampled at all of these sites  ART=antiretroviral therapy; CRP=C-reactive protein; Hb=Haemoglobin; IQR=Interquartile range; TB=Tuberculosis | | |


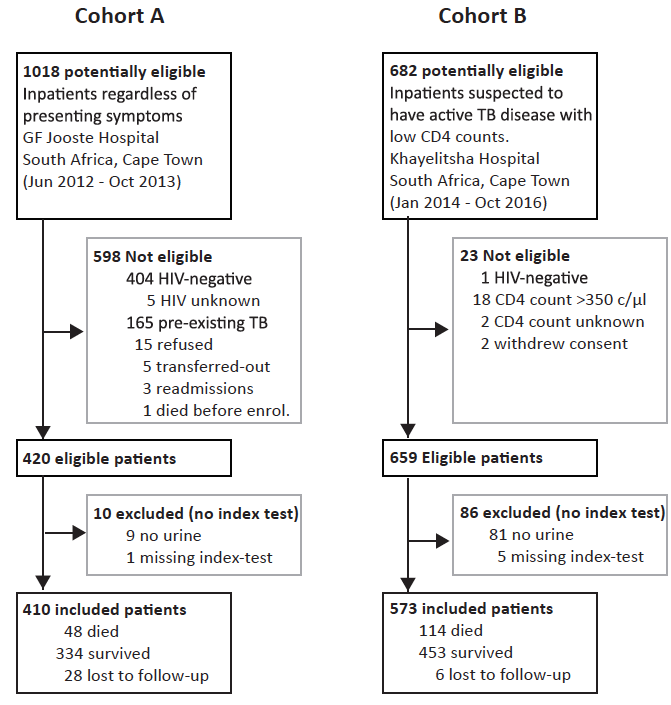


**Supplementary Figure 1:** Study flow


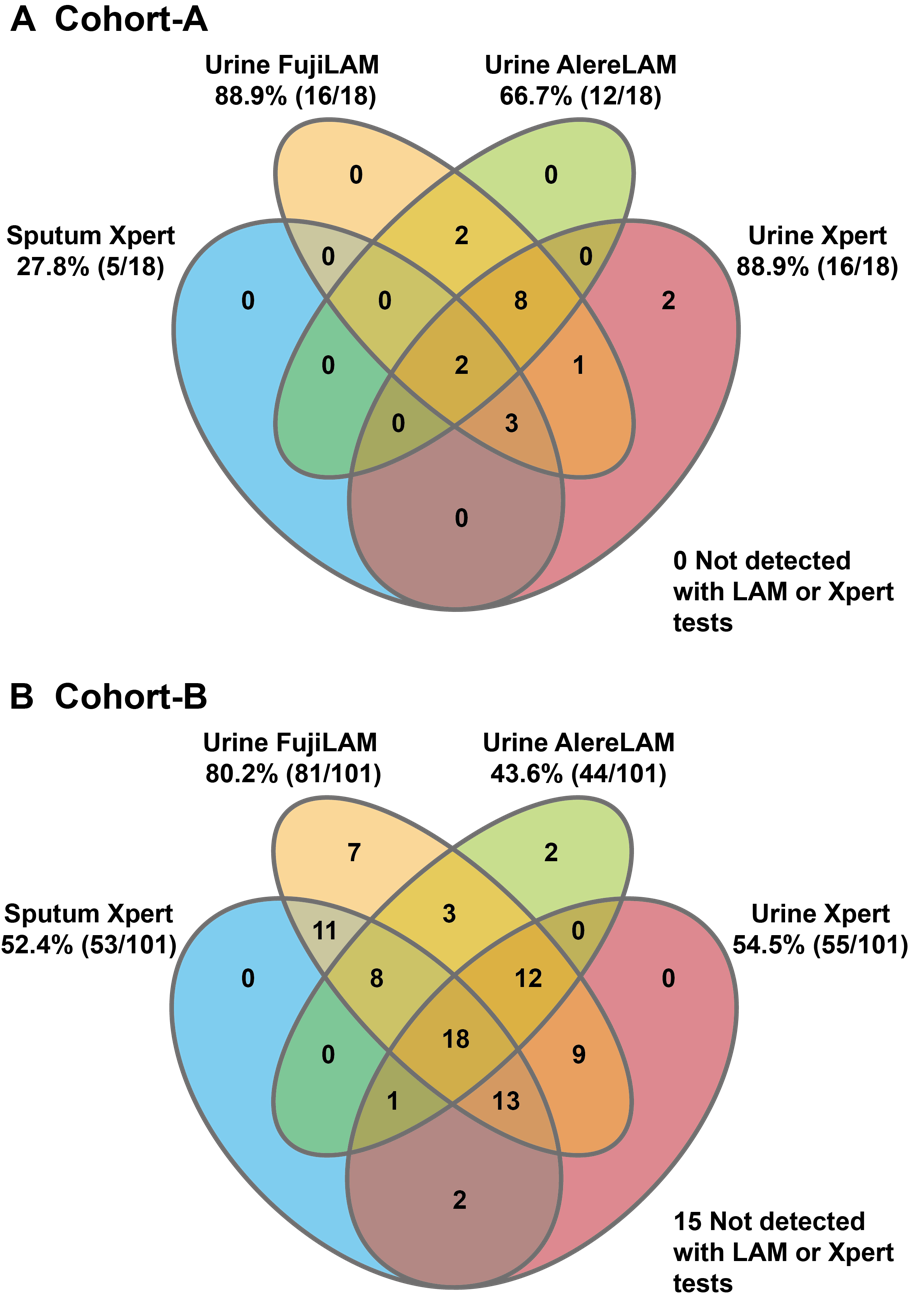

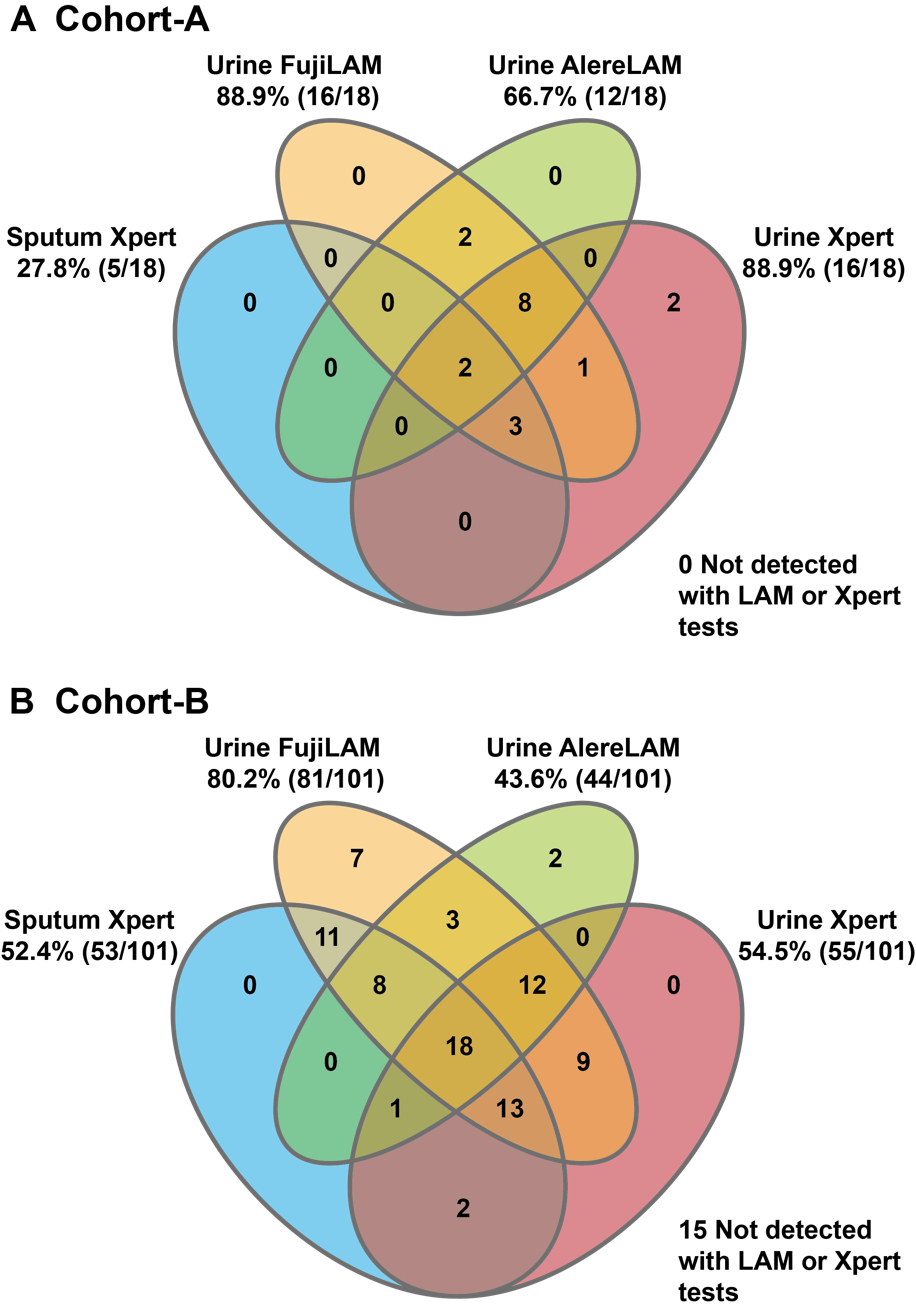


**Supplementary figure 2:** Diagnostic yield of LAM and Xpert assays in TB patients who died, per cohort.


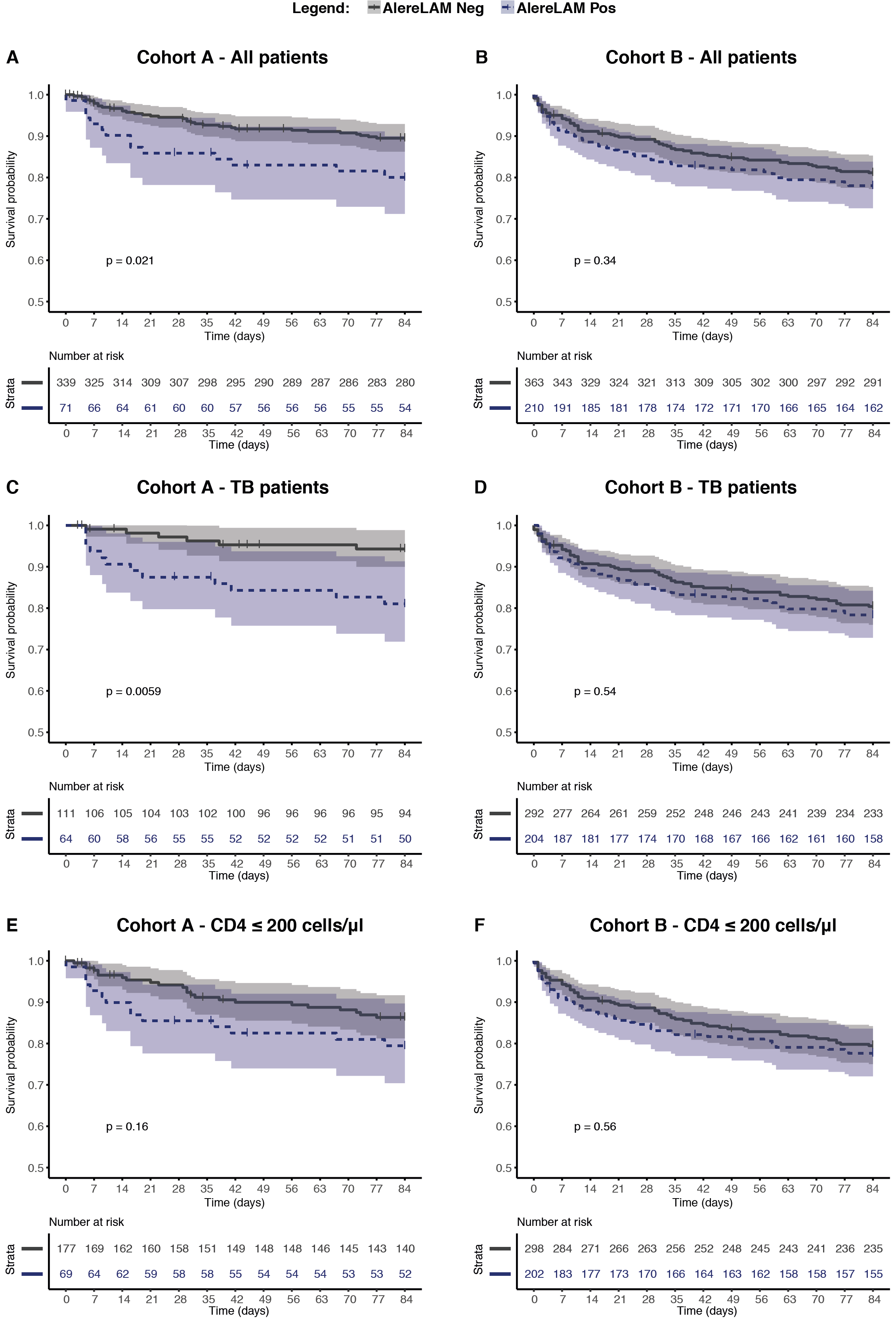


**Supplementary Figure 3:** Kaplan Meier survival curves by AlereLAM status, up to 12-weeks of follow up. All patients of Cohort-A (n=410) (Panel A) and Cohort-B (n=573) (**Panel B)**; TB patients from Cohort-A (n=175) (**Panel C)**, and Cohort-B (n=496) (**Panel D)**; Patients with CD4 ≤200 cells/µL, irrespective of TB diagnosis from Cohort-A (n=246) (**Panel E)** and Cohort-B (n=500) (**Panel F)**. p values are based on log-rank test and bands represent 95% confidence intervals.


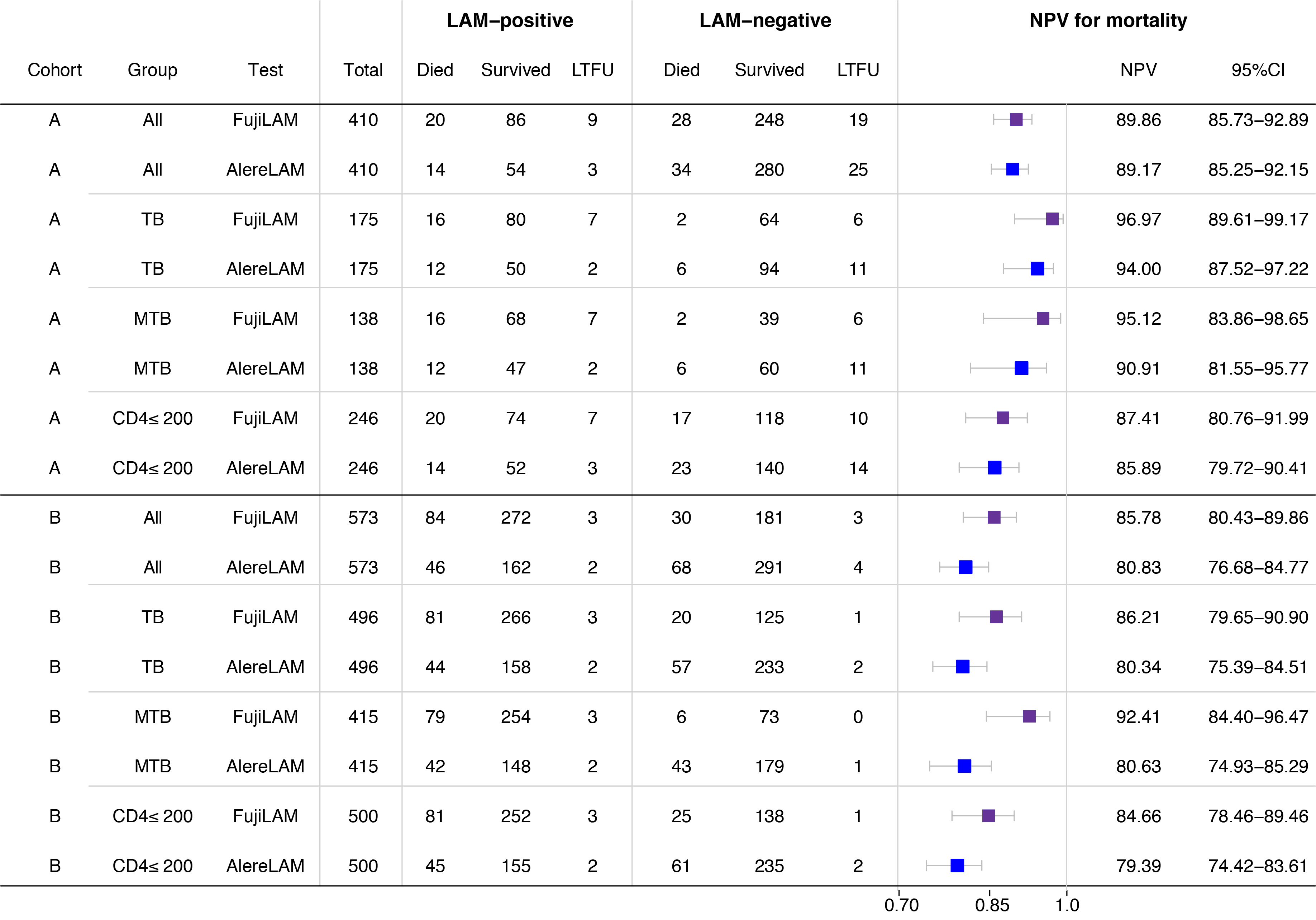


**Supplementary Figure 4:** Forest plots of Negative Predictive Value for mortality.

CD4≤200 cells/µL=All patients with CD4≤200 cells/µL, irrespective of TB diagnosis; CI=Confidence Interval; LTFU=Lost to follow-up; MTB=Microbiologically-confirmed TB; NPV=Negative Predictive Value; TB=Tuberculosis (which refers to both microbiologically- and clinically-confirmed Tuberculosis patients).
